# Cellular Robustness Conferred by Genetic Crosstalk Underlies Resistance against Chemotherapeutic Drug Doxorubicin in Fission Yeast

**DOI:** 10.1371/journal.pone.0055041

**Published:** 2013-01-24

**Authors:** Zoey Tay, Ru Jun Eng, Kenichi Sajiki, Kim Kiat Lim, Ming Yi Tang, Mitsuhiro Yanagida, Ee Sin Chen

**Affiliations:** 1 Department of Biochemistry, Yong Loo Lin School of Medicine, National University of Singapore, Singapore; 2 National University Health System, Singapore; 3 G0 Cell Unit, Okinawa Institute of Science and Technology, Okinawa, Japan; University of Cambridge, United Kingdom

## Abstract

Doxorubicin is an anthracycline antibiotic that is among one of the most commonly used chemotherapeutic agents in the clinical setting. The usage of doxorubicin is faced with many problems including severe side effects and chemoresistance. To overcome these challenges, it is important to gain an understanding of the underlying molecular mechanisms with regards to the mode of action of doxorubicin. To facilitate this aim, we identified the genes that are required for doxorubicin resistance in the fission yeast *Schizosaccharomyces pombe*. We further demonstrated interplay between factors controlling various aspects of chromosome metabolism, mitochondrial respiration and membrane transport. In the nucleus we observed that the subunits of the Ino80, RSC, and SAGA complexes function in the similar epistatic group that shares significant overlap with the homologous recombination genes. However, these factors generally act in synergistic manner with the chromosome segregation regulator DASH complex proteins, possibly forming two major arms for regulating doxorubicin resistance in the nucleus. Simultaneous disruption of genes function in membrane efflux transport or the mitochondrial respiratory chain integrity in the mutants defective in either Ino80 or HR function resulted in cumulative upregulation of drug-specific growth defects, suggesting a rewiring of pathways that synergize only when the cells is exposed to the cytotoxic stress. Taken together, our work not only identified factors that are required for survival of the cells in the presence of doxorubicin but has further demonstrated that an extensive molecular crosstalk exists between these factors to robustly confer doxorubicin resistance.

## Introduction

Doxorubicin (DOXO)(trade name adriamycin) is an anthracycline antibiotic that ranks among the most useful anti-neoplastic agents used against a wide range of cancers including that of breast, prostrate, oesophageal, stomach, liver; sarcomas and hematological malignancies [1], [2]. DOXO acts by inhibiting the DNA topology regulating enzyme topoisomerase II (Top2), which relieves torsional stress on the chromatin generated during DNA transactions such as transcription and DNA replication via transient formation of DNA double stranded breaks (DSB) [3], [4]. Poisoning of Top2 presumably results in accumulation of DSB that eventually leads to cell death [3], [5]. In combination with formaldehyde, DOXO can also form DNA interstrand crosslink-like adducts, which interfere with DNA replication [5]. Notwithstanding the effect on DNA, DOXO has been reported to disrupt mitochondrial membrane lipid cardiolipin and induce oxidative stress and production of reactive oxygen species [1], [2].

A major challenge in the usage of DOXO lies in the control of the administration dose as increase dosage heightens the propensity of adverse drug-linked cytotoxic effect, especially cardiotoxicity [1], [2]. Another problem in DOXO usage is the rapid development of resistance by the cancer cells [6]. The underlying molecular mechanisms causing drug resistance is still largely unresolved, which make the management of it difficult. Hence an understanding of the basic mechanisms employed by cells to resist the ill effects of DOXO is essential to fine-tune drug usage to achieve maximum killing of malignant cells yet minimizing cytotoxic side effects.

One way to address this issue is via identification and elucidation of the molecular players and pathways modulating DOXO resistance. We therefore embarked on an unbiased genetic screen using the model organism fission yeast *Schizosaccharomyces pombe* aiming to uncover fundamental mechanisms regulating DOXO resistance (DXR). We identified independent mutations in 91 DXR genes that were required for resistance against DOXO from a collection of 3225 single gene deletion mutants. These DXR factors function in multiple distinct complexes that acted in several sub-cellular locations. We have further shown that these DXR factors also cooperated to form an extensive network to maintain cell viability in the presence of DOXO. Hence the work reported here suggests that resistance to DOXO was controlled by a web of interlinking pathways and the knowledge derived are expected to be useful for facilitating selection of diagnostic targets for combination chemotherapy in conjunction with DOXO in human cells.

### Experimental Procedures

#### Strains, media and drugs

Fission yeast haploid gene deletion library (Bioneer ver2.0) [7] was used for genome-wide screens with the supplied wild-type and *Δrav1* [8] strains as negative and positive controls respectively. Strains were tested by growing to exponential phase followed by spotting individual ten-fold serially diluted strains manually on agar media containing DOXO and incubated at 26°C for three to four days. Prototrophic strains were derived from backcrossing auxotrophic Bioneer library strains with prototrophic 972 (*h^−^*) strain using classical genetic techniques. YEA (3% glucose, 0.5% yeast extract, 75 mg/L adenine) media was used to grow fission yeast cells. The gene disruption for each strain according to Bioneer was confirmed by checking for the replacement of endogenous open reading frame of each gene with the kanamycin disruption cassette using PCR.

#### Determination of DOXO hypersensitivity of mutant strains

DOXO hypersensitivity of the single mutants (SM) was determined by spotting prototrophic mutant strains at 75 µg/ml DOXO. Strains that did not show hypersensitivity were retested on 165 µg/ml, and further at 310 µg/ml. Strains which showed hypersensitivity at 75, 165, and 310 µg/ml DOXO were denoted as strong, medium and weak mutants respectively. Analysis of genetic interactions were performed by comparing the fold sensitivity (relative to wild-type (WT) cells) of the double mutant (DM) to that of the single mutants and then normalizing to the growth of the DM on media without drug. A DM strain was deemed to show synthetic growth defect if its relative fold hypersensitivity on DOXO was higher than both the parental SMs, whereas in the case of non-synthetic growth defect, the relative fold hypersensitivity of the DM was similar to the parental strain that exhibited higher fold hypersensitivity.

## Results

### Identification of Doxorubicin Resistance (DXR) Genes in Fission Yeast

**Figure 1:**
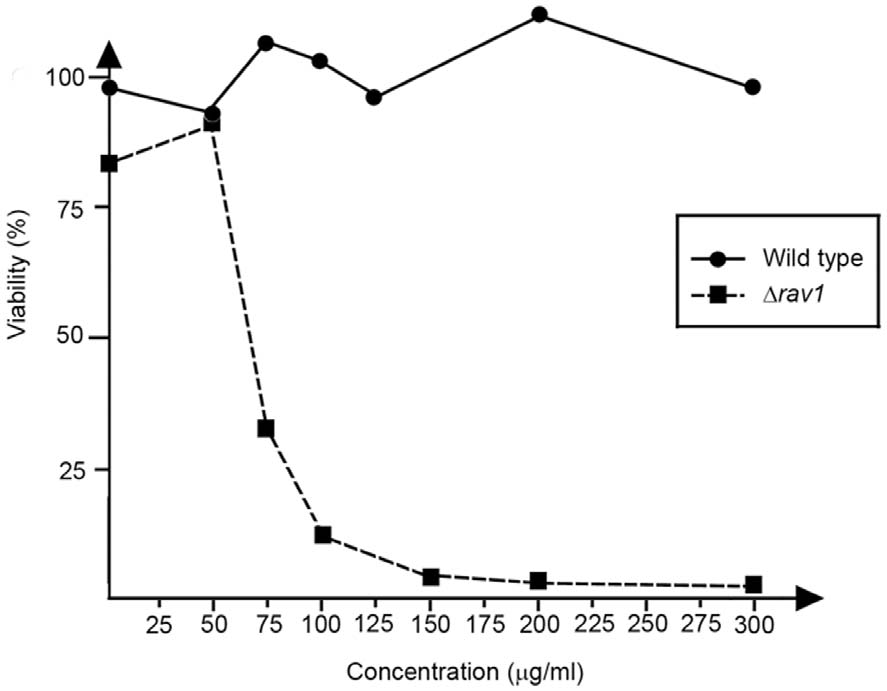
Cytotoxicity of wild-type and *Δrav1* cells to concentrations of DOXO ranging from 0 to 300 **µg/ml.** Exponentially-growing cells were treated with the indicated level of DOXO for 4 hours. Cell viability was estimated by the number of colonies that was formed after seven day incubation from 200 cells plated on rich media without drug, and expressed as a proportion to the untreated sample. Wild-type cells did not show decrease in viability over the range tested, while *Δrav1* showed >50% loss in viability at 75 µg/ml.

**Figure 2:**
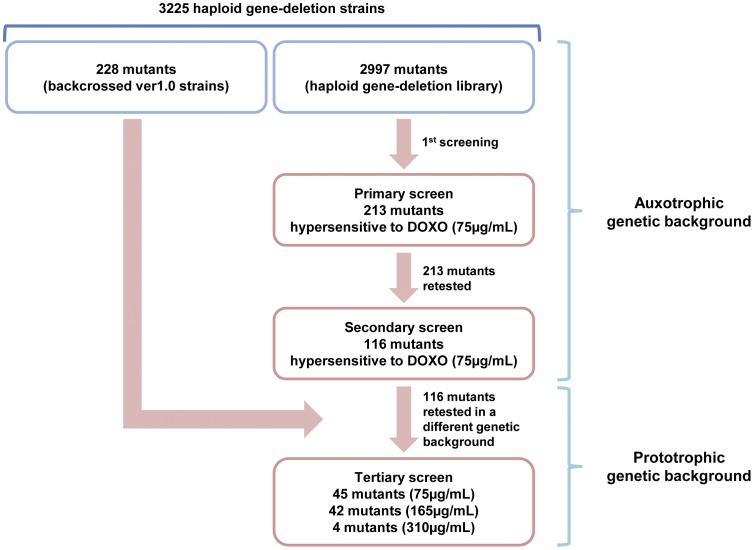
Workflow of the screening procedure to uncover genes required for DOXO resistance (DXR). A total of 3225 strains made up of 2997 strains from Bioneer ver2.0 library and 228 unique backcrossed strains from ver1.0 were screened. Each of the auxotrophic ver2.0 strains was serially diluted and manually spotted on 75 µg/ml DOXO and 116 strains were found to be repeatedly showing hypersensitivity on DOXO. These strains were backcrossed with prototrophic wild-type cells to remove all the nutrition marker mutations in the Bioneer strains in order to link the DOXO hypersensitive phenotypes to the indicated null mutations. 91 strains that showed hypersensitivity to various level of DOXO were obtained finally.

To find genes essential for chemoresistance against doxorubicin (DOXO), we began by examining the cytotoxicity of DOXO on fission yeast cell. This was achieved by determining the viability at a range of different DOXO concentrations (Fig. 1). Viability of wild-type cells remained high over the range tested until 300 µg/ml (Fig. 1) while a mutant of the gene encoding a regulator of assembly of V-ATPase (*Δrav1*) showed hypersensitivity to DOXO as reported before [8]. We chose 75 µg/ml (129 µM) that represented a >LC50 dose that killed >50% of *Δrav1* cells (Fig. 1) to search in an unbiased manner for strains that exhibited hypersensitivity to DOXO from among 3225 fission yeast single gene deletion strains. This collection consisted of 2997 strains from Bioneer ver2.0 library and 228 strains from ver1.0 but absent in ver2.0 library [7] (Fig. 2). The ver1.0 strains have been backcrossed with wild-type strain to obtained prototrophic mutants.

**Figure 3:**
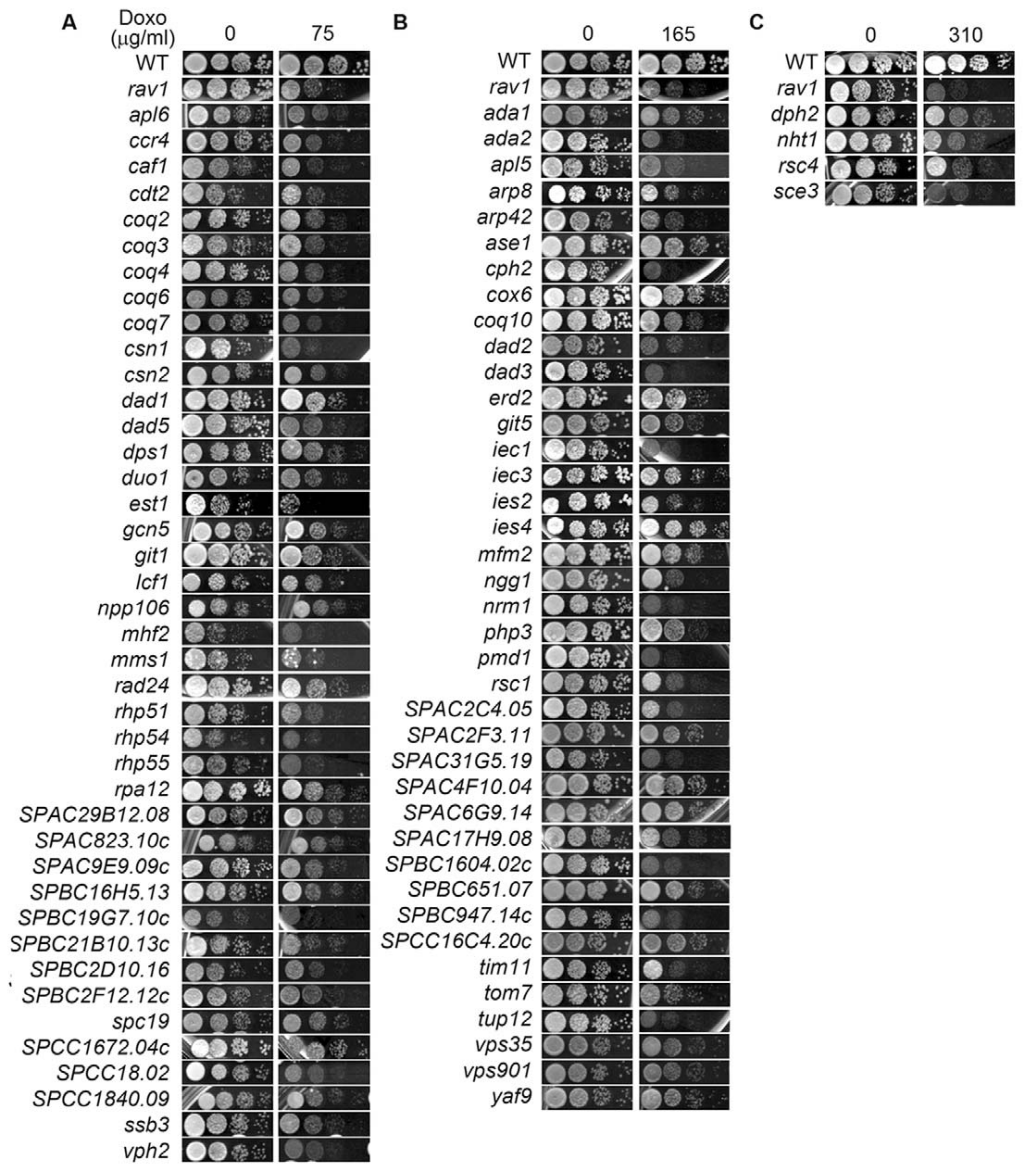
Prototrophic DXR haploid deletion strains showed differential sensitivity to DOXO. Hypersensitivity demonstrated by DXR strains at (A) 75 µg/ml, (B) 165 µg/ml and (C) 310 µg/ml DOXO. Exponentially growing prototrophic strains were ten-fold serially diluted and individually spotted on DOXO-containing plates.

The strains were tested by manually spotting ten-fold serially diluted exponentially growing cells on solid media containing 75 µg/ml DOXO. Repeatability of the DOXO-hypersensitive phenotype of the strains was confirmed by spotting the strains at least four times on DOXO-containing agar media. Furthermore, we also performed backcross to introduce the gene deletion mutations into a prototrophic background so as to ascertain that the DOXO hypersensitivity was specific to the null mutation and unaffected by the genetic background of the strains (Fig. 2). Of the 116 DXR strains obtained from the primary and secondary screens, 91 strains remained hypersensitive to DOXO in prototrophic background (Fig. 2). The remaining 27 null mutations failed to show DOXO hypersensitivity in the prototrophic genetic background indicating that the phenotype may arise from genetic interaction between the nutrition marker gene mutations and the respective gene deletions (Fig. 2). These strains were hence excluded from further analysis. The viability loss demonstrated by these 91 hypersensitive strains showed that the deleted genes from each of the strains were required for conferring DOXO resistance (DXR) to the cells. These mutants were shown to be sensitive at varying concentrations of DOXO (Fig. 2, Table S1) with 45 strongly, 42 medium and 4 weakly sensitive strains, showing hypersensitivity at 75 µg/ml, 165 µg/ml and 310 µg/ml DOXO respectively (Fig. 3A, B, C, S1, Table S1). Several strains exhibited considerable growth defect already in the absence of the drugs (Fig. S1). However these were still categorized as DXR strains due to the additive growth defect on exposure to the drug (Fig. S1). We noticed that most of the DXR genes possess counterparts in budding yeast and a significant number (52 out of 91, 57.1%) showed high similarity to human proteins (Table S1) as identified using Homologene (NCBI) and Pombase (Sanger Center) databases.

### Functional Analysis of DXR Genes

**Figure 4:**
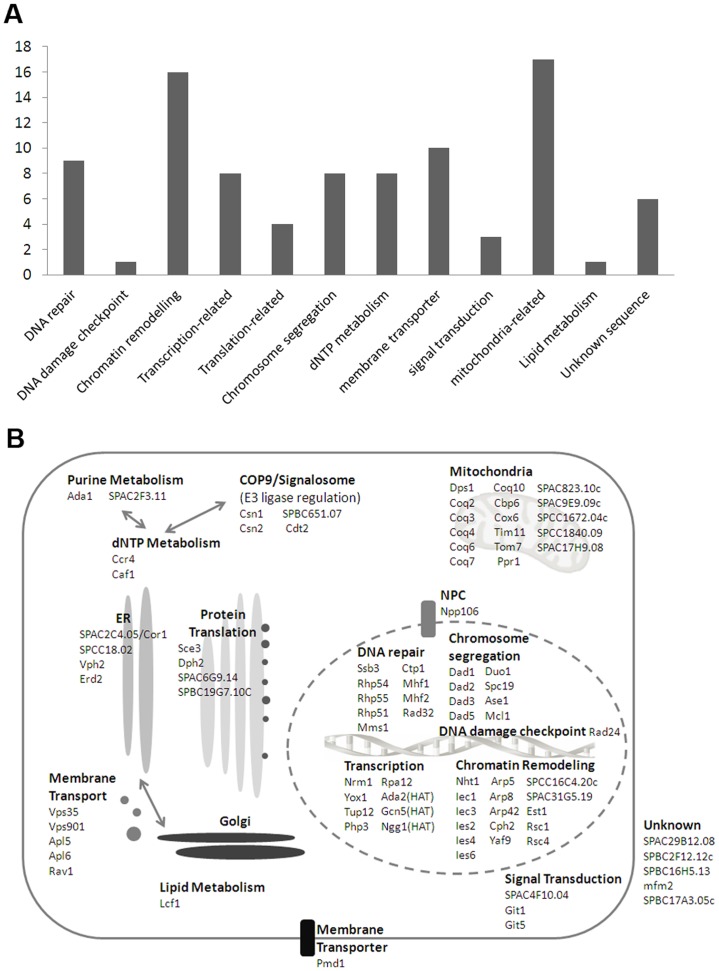
Functional classification and sub-cellular localization of DXR factors. (A) DXR factors were classified into 11 ontological groups. Vertical axis indicates the number of genes in each class. (B) Model of molecular mechanism for DOXO resistance in fission yeast. Cooperation of chromosome associated complexes that remodel and coordinate proper response to DNA damage were probably required for precise management of DOXO-induced DNA lesions. In addition, mitochondrial function and membrane transporters localized to various cell compartments that control intracellular accumulation of DOXO also determined the sensitivity of cells to the drug. Several factors including the COP9-Signalosome may facilitate repair of DOXO lesion via regulation of nucleotide synthesis.

Next we used Gene Ontology Local Exploration Map (GOLEM) (http://go.princeton.edu/cgi-bin/GOTermFinder) to classify the DXR genes into different functional categories. At a statistical confidence of p<0.01, GOLEM allocated 44 genes to nuclear processes related to chromatin remodeling, histone modification (acetylation in particular), chromosome structure maintenance and segregation, homologous recombination (HR) and DNA repair pathways (Fig. S2). Six genes were classified in mitochondrial respiration pathways by GOLEM (Fig. S3), which also identified many genes encoding membrane associated transporter proteins (Table S1). Coupling with published data and information from PomBase (Sanger Center), we were able to divide the DXR mutants into 11 physiological pathways (Table S1, Fig. 4A). Approximately 80% of the DXR factors localized to three subcellular compartments, namely nucleus (43 of 91, 47.3%), mitochondria (17 of 91, 18.7%) and endosomal membranes (11 of 91, 12.1%) (Fig. 4B).

Several distinct complexes were identified among the nuclear DXR factors that include Ino80 (Nht1, Iec1, SPCC16C4.20c, Ies6, Iec3, Ies4, Arp5, Arp8) [9], RSC chromatin remodelers (Arp42, Rsc1, Rsc4) [10], SAGA histone acetyltransferase complex (Gcn5, Ngg1, Ada2) [11], and DASH complex that controls microtubule-kinetochore attachment (Duo1, Spc19, Dad2, Dad3, Dad5) [12]. We also found many mutants of enzymes catalyzing biosynthesis of coenzyme Q, a major anti-oxidant that functions in mitochondria as electron transducer (*coq2*, *coq3*, *coq4*, *coq6*, *coq7,* and *dps1*) [13] (Fig. S3), as well as mutants of several subunits of the mitochondrial respiratory chain complexes (Pombase)(Fig. 4B, Table S1).

### Crosstalk between DXR Genes Confers Cellular Robustness in Doxorubicin

We performed epistasis analyses to assess the actual functional link between the DXR factors. To this end, we combined the null mutations together using genetic crosses and examined the hypersensitivity of the resultant double mutants (DM) relative to the corresponding single mutants (SMs). Cumulative hypersensitivity shown by the DM over the SM indicates that the factors function in separate biological pathways. In contrast, for factors interacting with each other in the similar pathways, the DM would be expected to exhibit no cumulative DOXO hypersensitivity [14]. We concentrated our studies on the factors classified under the three major ontological components, namely nuclear chromatin, mitochondrial and endosomal regulation.

Epistasis analyses were carried out between mutants of the subunits within the Ino80 and DASH complexes (Ino80: *Δnht1*, *Δspcc16c4.20c* and *Δiec1*, and DASH: *Δdad2*, *Δdad3* and *Δdad5*) (Table S1). The lack of synthetic hypersensitivity exhibited by the DMs on DOXO compared to the SMs suggests that the whole complexes may be important for DOXO response and the subunits likely did not reorganize into different synergistically-acting sub-complexes (Fig. S4, S5). We showed that the two HR factors Rhp54 and Rhp55 also function in the same complementation group similar to the response exhibited towards several other DNA damaging agents [15]. The positive interaction exhibited by these two HR mutants on DOXO was suggestive of a closer relationship that may involve physical interaction [16]. Interestingly, we found that Ssb3, the single stranded DNA binding factor that facilitates repair of DNA replication-associated damage [17] also belonged to the same epistatic pathway as Rhp55 (Fig. S6). However, *Δssb3* showed synthetic growth defect with *Δrhp54*, suggesting that there exists subdivision of pathways within the HR group proteins in respond to DOXO (Fig. S6).

**Figure 5:**
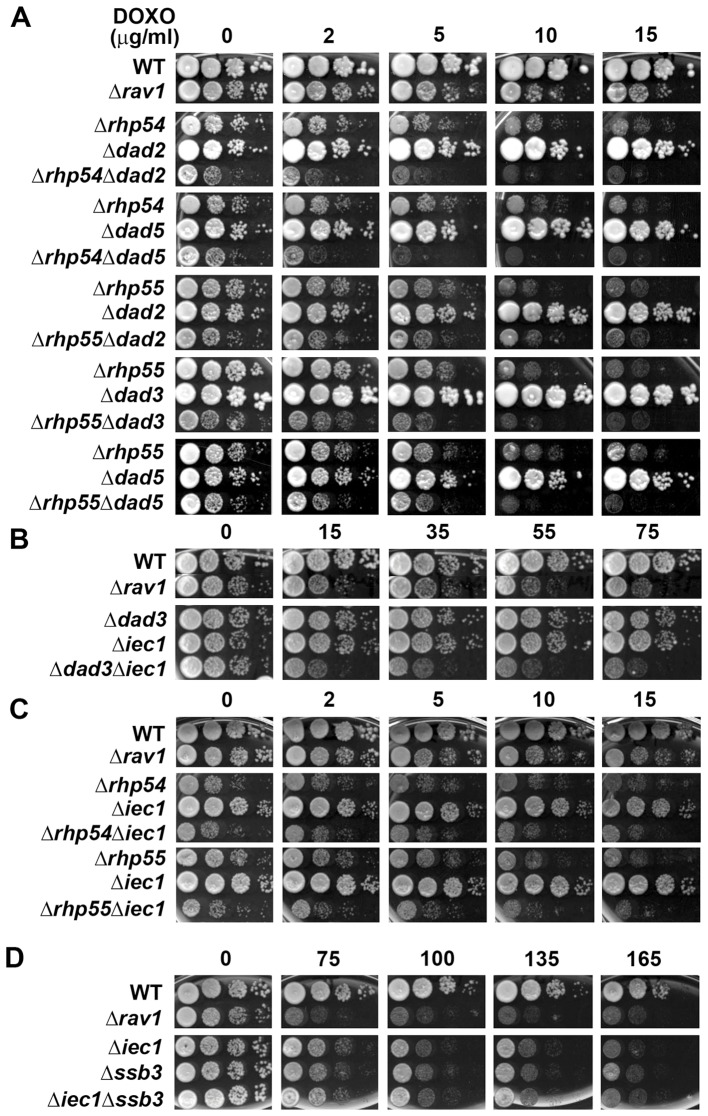
Functional crosstalk shown by the genetic interaction between nuclear DXR factors. (A) Genetic interaction between mutants of DASH complex (*Δdad2*, *Δdad3*, and *Δdad5*) and HR genes (*Δrhp54* and *Δrhp55*). (B) Mutant of Ino80 subunit *Δiec1* showed synthetic negative effect with that of DASH mutant *Δdad3*. (C) Ino80 mutant *Δiec1* showed no synthetic growth defect with the HR mutants *Δrhp54* and *Δrhp55* suggesting that Ino80 function in the same pathway with HR genes to modulate resistance to DOXO. (D) Lack of synthetic growth defect between Ino80 mutant *Δiec1* and that of the single-stranded DNA binding protein Ssb3.

Next we tested the genetic interaction between several nuclear-localizing complexes, namely DASH, Ino80, SAGA, RSC and the HR proteins [9], [10], [11], [15], [18]. We observed synthetic growth defects between Ino80 and HR factor mutants with that of DASH subunits (*Δrhp54Δdad5* and *Δrhp55Δdad5,* weaker effect in *Δrhp54Δdad2* and *Δrhp55Δdad3*), suggesting that the chromatin-localizing Ino80 and HR proteins may cooperate in parallel with DASH complex, which regulates precise chromosome segregation [12] to confer DOXO resistance in fission yeast cells (Fig. 5A and 5B). Consistent with this hypothesis, mutants of Ino80 and HR factors showed no cumulative increase in DOXO hypersensitivity with the mutants of other chromatin acting complex subunits (SAGA, Fig. S7 and RSC, Fig. S8). Furthermore, no DOXO-dependent synthetic growth defect was observed between mutants of the Ino80 subunit *iec1* and single stranded DNA binding Ssb3, which is in the same epistasis group as Rhp55 (Fig. 5D, S6). Taken together, these epistasis studies suggest that the HR, Ino80, SAGA and RSC proteins may function closely to confer DOXO resistance, possibly by coordinating the repair of DOXO-induced DNA aberration. Interestingly, we observed that *Δdad2* SM showed non-synthetic interaction with *Δrhp55* SM (Fig. 5A), suggesting that there may exist yet undefined functional overlap between the DASH complex and Rhp55.

**Figure 6:**
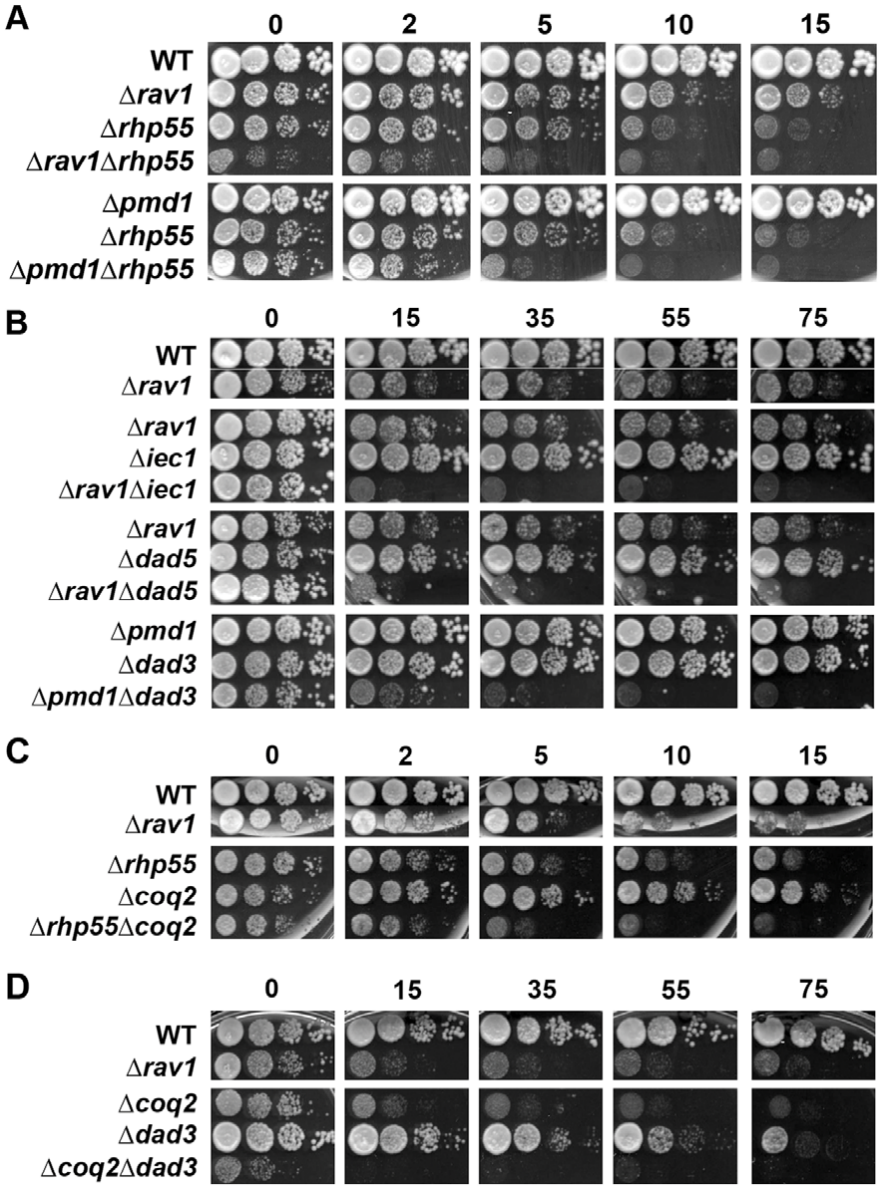
Synergistic effect between the nuclear, mitochondrial and membrane transport pathways to counteract DOXO cytotoxicity. Genetic interaction between the genes encoding membrane transporters (*Δrav1* and *Δpmd1*) and (A) HR (*Δrhp55*), (B) Ino80 (*Δiec1*), and DASH subunits (*Δdad3* or *Δdad5*). Genetic interaction between mitochondrial coenzyme Q biosynthesis enzyme *(Δcoq2*) and (C) HR (*Δrhp55*), and (D) DASH subunit (*Δdad3*). All of the mutant pairs showed prominent synthetic growth defect, except *Δrhp55Δrav1* of which the synthetic growth defect was weak and masked by the strong drug-independent growth defect of the DM.

**Figure 7:**
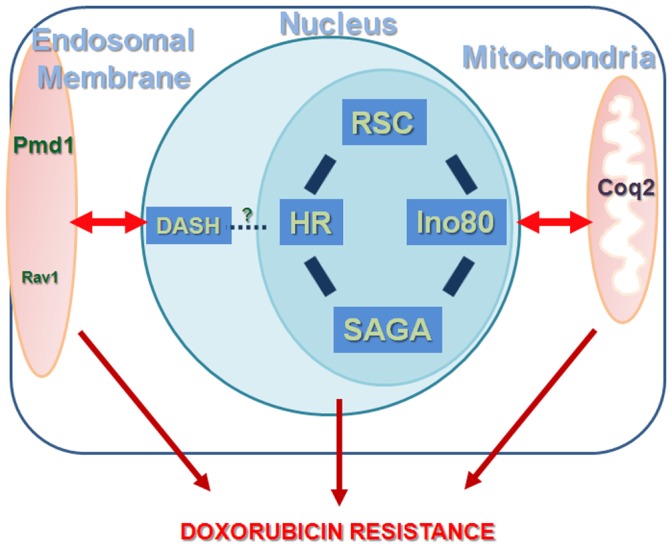
Diagrammatic representation that depicts synergistic relationship between the nuclear, mitochondrial and endosomal membrane transporter genes. Lines with double arrow heads represent synthetic growth defect exhibited by the double mutants in different subcellular locations indicative of a synergistic relationship between components at these locations. Within the nucleus, chromatin modulating factors that act in similar epistatic group are joined by bold lines and these factors generally function in parallel to the DASH complex (dotted line). ‘?’ indicates undefined link between DASH and HR (Rhp55) factors. The composite crosstalk between the factors acting within and between each cellular compartment contributes to the resistance against DOXO in fission yeast.

In addition, further analyses showed that the above-mentioned nuclear factors functioned distinctly from the membrane transporters (Pmd1 and V-ATPase regulator Rav1, the synthetic effect with *Δrav1* was weaker) (Fig. 6A, 6B), and mitochondrial pathway involving coenzyme Q biosynthesis (Coq2)(Fig. 6C, 6D) as the DMs were more sensitive to DOXO than the corresponding SMs. Taken together, our results suggest that there exist two major mechanisms, one dependent on DASH complex and the other on Ino80/HR/RSC/SAGA that worked in sync to counteract the cytotoxic effect of DOXO. In the meantime, these nuclear factors were integrated into a larger network that involved synergistically acting mitochondrial ATP synthesis factors and membrane transporter proteins, thus conferring DOXO resistance (Fig. 7).

## Discussion

Here we report the identification of genes constituting an extensive network regulating resistance against the chemotherapeutic drug doxorubicin (DOXO). Such genetic crosstalk occurred between many macromolecular complexes in several sub-cellular locations, in particular nucleus, mitochondria and endosomal membranes, which cooperate to ensure cell viability when challenged with the cytotoxic drug. Our analyses hence emphasized the importance of interplay between molecular factors in the control of chemotherapeutic drug response, which is likely to be a universal mechanism controlling the resistance against chemotherapeutic drugs.

Redundancy between cellular pathways has been proposed to confer robustness of cells to external perturbation [19], [20]. Consistent with this model, we observed that prominent cell death in DOXO seldom resulted from disruption of a single DXR gene but simultaneous abrogation of two or more factors was required. Interestingly, we noticed several DOXO-specific genetic interactions between DXR genes that were not observed in the absence of the drug. This suggests that cellular pathways can be dynamically “rewired” when challenged with DOXO. Such capability to remodel their genetic connectivity possibly underlies the flexibility that enable cells to respond only when disturbances are present, thus optimizing the utilization of cellular resources to achieve maximum efficiency of the living system [21].

As compared to a similar screen that isolated DOXO-hypersensitive strains from the budding yeast, there were several overlapping groups of DXR genes with the screen reported here [22]. Among these were genes encoding several HR protein including SpRhp51/ScRAD51, SpRhp55/ScRAD55, SpRad32/ScMre11 (*S. pombe* (Sp) and *S. cerevisiae* (Sc)) [23]. Mutants of these genes were among the most sensitive to DOXO, strongly suggesting that HR-regulated DNA repair pathway is a conserved response to resolve DOXO-induced DNA adducts in both yeasts. Besides HR, genes encoding protein subunits of the SAGA, RSC and Ino80 complexes and that in endosomal sorting pathway were also identified [10]–[12], [22]. The major differences between the genes obtained from the two screens were the membrane transporter group that include fission yeast homologue of human P-gp, the chromosome segregation regulator DASH complex and the anti-oxidant coenzyme Q biosynthesis genes [6], [12], [13], which were found as major hits in our list but were not isolated from budding yeast. In terms of specific genes, there was surprisingly low degree of overlap between the two yeasts (seven out of 71 genes obtained by Xia *et al.* [22]). However more similarity was observed when the DOXO-responsive molecular pathways were considered.

Multiple mutants of the Ino80 complex subunits exhibited hypersensitivity to DOXO were obtained in our screen. Ino80 is a member of the SWI/SNF family ATP-dependent chromatin-remodeling complex that functions in DNA DSB repair and transcription from yeast to human [9], [23]–[25]. Interestingly, our genetic analysis observed no (or very marginal) upregulation of DOXO hypersensitivity in double mutants of Ino80 subunit (*Δiec1*), the HR genes *(Δrhp54*, *Δrhp55*) and *Δssb3*, showing that Ino80 complex and the HR proteins probably function in the same pathway. In addition, we also observed no synthetic defect between the mutant of SAGA catalytic subunit (*Δgcn5*) and that of HR or Ino80 complex, suggesting that all these machineries closely cooperate to mediate repair of DOXO-induced lesions, possibly in the recruitment of DNA damage response factors.

It is not absolutely clear regarding the nature of the lesions arise from DOXO exposure but the drug has been associated with formation of DNA double-stranded break (DSB) [3] and DNA interstrand crosslink-like aberrations that can also result in DSB when the replication fork clashes into such structure [5]. Homologous recombination (HR) pathway is essential for the repair of DOXO-associated DNA lesions in both scenarios [5]. It is therefore not surprising that our screen and that in budding yeast [22] identified mutations in HR genes to result in DOXO hypersensitivity. Interestingly, our results identified the Rpa3-like single stranded DNA binding protein Ssb3 functioning in the same epistasis group as the HR protein Rhp55. Ssb3 is required for repair of DNA lesions during S phase of the cell cycle [17], supporting the hypothesis that DOXO induced chromosomal conformation change that impede the progress of the replication fork. Furthermore, we also identified human MHF1- and MHF2-like proteins, which interact and facilitate the Fanconi anemia nuclear core complex in resolving branched DNA structure including replication fork [26], further supporting the formation of DNA adducts that resulted in replication fork stalling in DOXO. Together with the epistatic relationship between the SWI/SNF ATP-dependent chromatin remodeling Ino80, SAGA histone acetyltransferase and RSC complexes with the HR proteins, we propose a model in which nucleosome remodeling and establishment of opened chromatin conformation via histone acetylation facilitate repair of DOXO adducts by HR factors and Ino80 complex probably during S-phase.

Remarkably, we isolated several enzymes participating in the biosynthesis of coenzyme Q/ubiquinone, which is a strong anti-oxidant [13] essential for maintaining the cell viability in the presence of DOXO in fission yeast. Coenzyme Q is the major electron carrier in the mitochondrial electron transport chain relaying electron between NADH dehydrogenase (complex I) and succinate dehydrogenase (complex II) to CoQ:cytochrome c reductase (complex III) [13]. In the human cells, proper mitochondrial function is important for resistance against DOXO and the increased inhibition of mitochondrial oxidative phosphorylation that leads to attenuation of ATP production by DOXO has been suggested to underlie DOXO dose-dependent cardiotoxicity [27]. In accordance with the anti-oxidant effect, coenzyme Q is sold as a dietary supplement in the market and the intake has been deemed to be beneficial for human heart function and to reduce the risk of cardiotoxicity during chemotherapeutic regiments [28], [29]. Our results throw a cautionary note to the consumption of coenzyme Q supplement during chemotherapy, as the extrapolation of that to the human cells suggest that coenzyme Q may also aid in the development of drug resistance by the cancer cells.

The development of drug resistance is a big hurdle that challenges the efficacy of chemotherapeutic treatment of cancers [6]. One major mechanism that contributes to drug resistance of the cancer cells is the upregulation of efflux transporter activity that results in extrusion of drug by the cancer cells [6]. Permeability-glycoprotein (P-gp) is an ABC type drug efflux transporter that commands great clinical interest and is the major transporter involved in extrusion of doxorubicin from the cell [30], [31], [32]. Overexpression of P-gp is closely associated with the development of multidrug resistance in the cancer patients [33]. Much effort has been devoted to targeting of P-gp to reverse multidrug resistance during chemotherapy but with marginal success [6], [31]. Our results here provide evidence that one main reason for this setback may lie in the existence of a complex interaction between redundant pathways that act together with the membrane transporters P-gp. The presence of such crosstalk will pose a difficulty for pharmaceutical intervention against specific efflux transporters. However, the definition of such molecular crosstalk may facilitate the identification of diagnostically important target proteins for pharmacological intervention, which may be useful for counteracting drug resistance in the human cells.

### Conclusions

Here we report the identification of 91 genes that were required for resistance against the anti-cancer drug doxorubicin in fission yeast. These genes include chromatin remodelers and chromosome segregation complexes that genetically cooperate with one another in the nucleus, which also synergize with components of electron transport chain in the mitochondria and efflux transporters on endosomal membrane. Such extensive redundancy was deemed to promote cellular robustness in the presence of the drug. The observed network may be used as a basis to study and improve the efficacy of doxorubicin usage via targeted intervention to sensitize human cancer cells to the drug.

## Supporting Information

Figure S1 **DOXO-hypersensitive strains that showed significant retarded growth in the absence of the drug.** Several strains identified from our screen to show hypersensitivity to DOXO were classified as strong/medium sensitive strains according to the drug levels at which they were sensitive at. However, these mutants were already showing much reduced growth retardation on medium without drug, with only two of the most concentrated spots grown. These mutants include (A) *Δctp1*, *Δies6*, and *Δrad32* that showed hypersensitivity at 75 µg/ml DOXO and (B) *Δarp5*, *Δmcl1* and *Δspbc17a3.05c* that were sensitive at 165 µg/ml DOXO.
(TIF)

Figure S2 **Ontological classification of DXR genes generated using GOLEM.** All highly significant DXR genes were allocated by GOLEM to pathways related to regulation of DNA damage response, homologous recombination, chromosome structure maintenance and chromosome segregation, chromatin remodeling and histone acetylation. The genes depicted in this chart were enriched in several distinct macromolecular complexes, namely the microtubule connector at kinetochore DASH (Duo1, Spc19, Dad1, Dad2, Dad3, Dad5), chromatin remodeler Ino80 (Nht1, SPCC16C4.02, Iec1, Ies2, Iec3, Ies4, Ies6, Arp5, Arp8), chromatin remodeler RSC (Rsc1, Rsc4, Arp42), SAGA (Gcn5, Ngg1, Ada2) and several HR factors (Rhp51, Rhp54, Rhp55).
(TIF)

Figure S3 **Coenzyme Q biosynthesis genes were essential for DOXO resistance in fission yeast.** Coq2, Coq4, Coq6, Coq7 and Dps1 were identified by GOLEM to function in the ubiquinone/coenzyme Q biosynthetic pathway.
(PDF)

Figure S4 **Genetic interaction between components of the Ino80 complex.** Mutants of the Ino80 complex subunits showed no cumulative sensitivity to DOXO in double mutant combination over single mutants suggesting that they function in the same complex to regulate DOXO resistance. (A) Single and double mutants between Iec1, Spcc16c4. 20c and Nht1 were ten-fold serially diluted and spotted on plates incorporated with the indicated concentrations of DOXO. (B) Schematic representation of the close relationship between Iec1, Spcc16c4. 20c and Nht1. Double arrowhead lines depict no synthetic effect.
(TIF)

Figure S5 **Genetic interaction between subunits of the DASH complex.** Lack of cumulative DOXO hypersensitivity between mutants of different subunits of the DASH complex. (A) Single and double mutants between Dad2, Dad3 and Dad5 were ten-fold serially diluted and spotted on plates incorporated with the indicated concentrations of DOXO. (B) Schematic representation of the close relationship between Dad2, Dad3 and Dad5. Double arrowhead lines depict no synthetic effect.
(TIF)

Figure S6 **Genetic interaction between homologous recombination DXR genes.** Genetic interaction between the single stranded DNA binding protein Ssb3 with the HR proteins Rhp54 and Rhp55 in DOXO. (A) Rhp54 and Rhp55 showed positive genetic interaction. Ssb3 was epistatic with Rhp55 suggesting that Ssb3 function in the same pathway with Rhp55. On the other hand, *Δssb3Δrhp54* showed cumulative hypersensitivity over the single mutants. (B) Schematic representation of the close relationship between Ssb3, Rhp54 and Rhp55. Double arrowhead lines depict no synthetic effect. Single arrowhead represents synthetic suppression with the mutant pointed by the arrow being suppressed. Double blunt ended line represents synthetic growth defect.
(TIF)

Figure S7 **Homologous recombination factors, SAGA and Ino80 complex subunits formed a single epistatic group.** (A) Mutants of the SAGA complex (*Δgcn5*), Ino80 complex (*Δiec1*) and homologous recombination factor (*Δrhp55*) were serially diluted and then manually spotted onto agar media containing the indicated concentrations of DOXO. *Δgcn5Δiec1* double mutant showed no cumulative hypersensitivity relative to single mutants, indicating that these components of the three complexes were in the similar epistasis group. (B) DOXO hypersensitivity of *Δrhp55Δgcn5* was equivalent to *Δrhp55*, which is the weaker of the two single mutants at the level of DOXO tested, showing that Rhp55 function in the similar epistatic group with Gcn5. (C) Schematic representation showing lack of synthetic growth defect (double arrowhead lines) between the mutants of SAGA (*Δgcn5*), Ino80 (*Δiec1*) and HR(*Δrhp55*) subunits.
(TIF)

Figure S8 **Homologous recombination, chromatin remodeler RSC complex and Ino80 complex subunits formed a single epistatic group.** (A) Mutants of the SAGA complex (*Δrsc4*), Ino80 complex (*Δiec1*) and homologous recombination factor (*Δrhp55*) were tested as in Fig. S8. *Δrsc4Δrhp55* and *Δrsc4Δiec1* double mutant showed no cumulative hypersensitivity relative to single mutants. (B) Schematic representation of the relationship between Rsc4, Iec1 and Rhp55. Double arrowhead lines represent epistatic interaction accompanied by no cumulative increase in DOXO hypersensitivity relative to the single mutants.
(TIF)

Table S1 **DXR factors from fission yeast, budding yeast and human.**
(DOC)
