# Supplementary figures and images for: Cellular Robustness Conferred by Genetic Crosstalk Underlies Resistance against Chemotherapeutic Drug Doxorubicin in Fission Yeast
Source: PLoS One. 2013 Jan 24;8(1):e55041. doi: 10.1371/journal.pone.0055041 (PMC3554685; doi:10.1371/journal.pone.0055041)

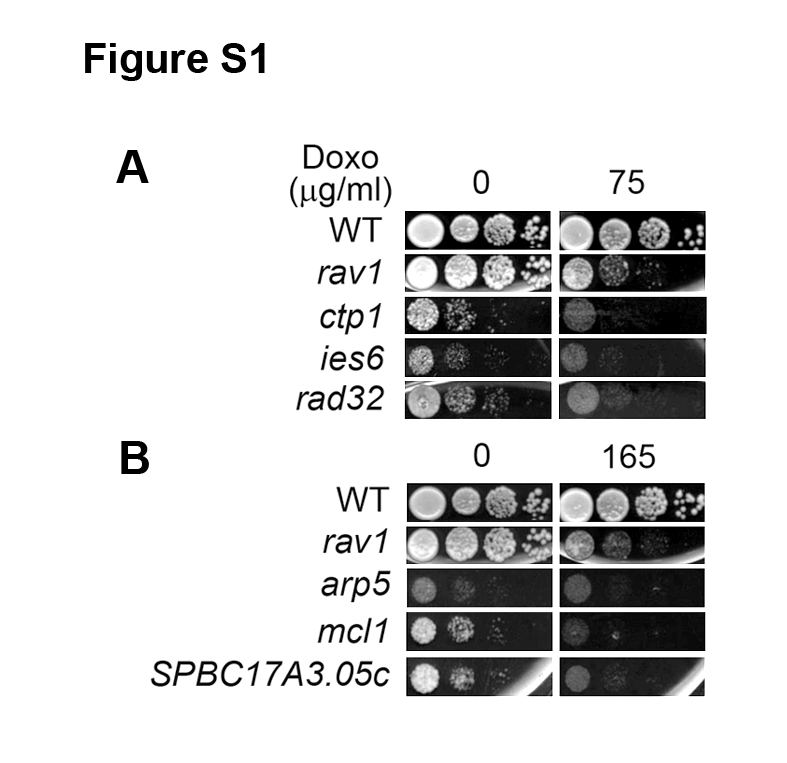

Supplement: Figure S1 — DOXO-hypersensitive strains that showed significant retarded growth in the absence of the drug. Several strains identified from our screen to show hypersensitivity to DOXO were classified as strong/medium sensitive strains according to the drug levels at which they were sensitive at. However, these mutants were already showing much reduced growth retardation on medium without drug, with only two of the most concentrated spots grown. These mutants include (A) Δctp1, Δies6, and Δrad32 that showed hypersensitivity at 75 µg/ml DOXO and (B) Δarp5, Δmcl1 and Δspbc17a3.05c that were sensitive at 165 µg/ml DOXO. (TIF) [file pone.0055041.s001.tif]

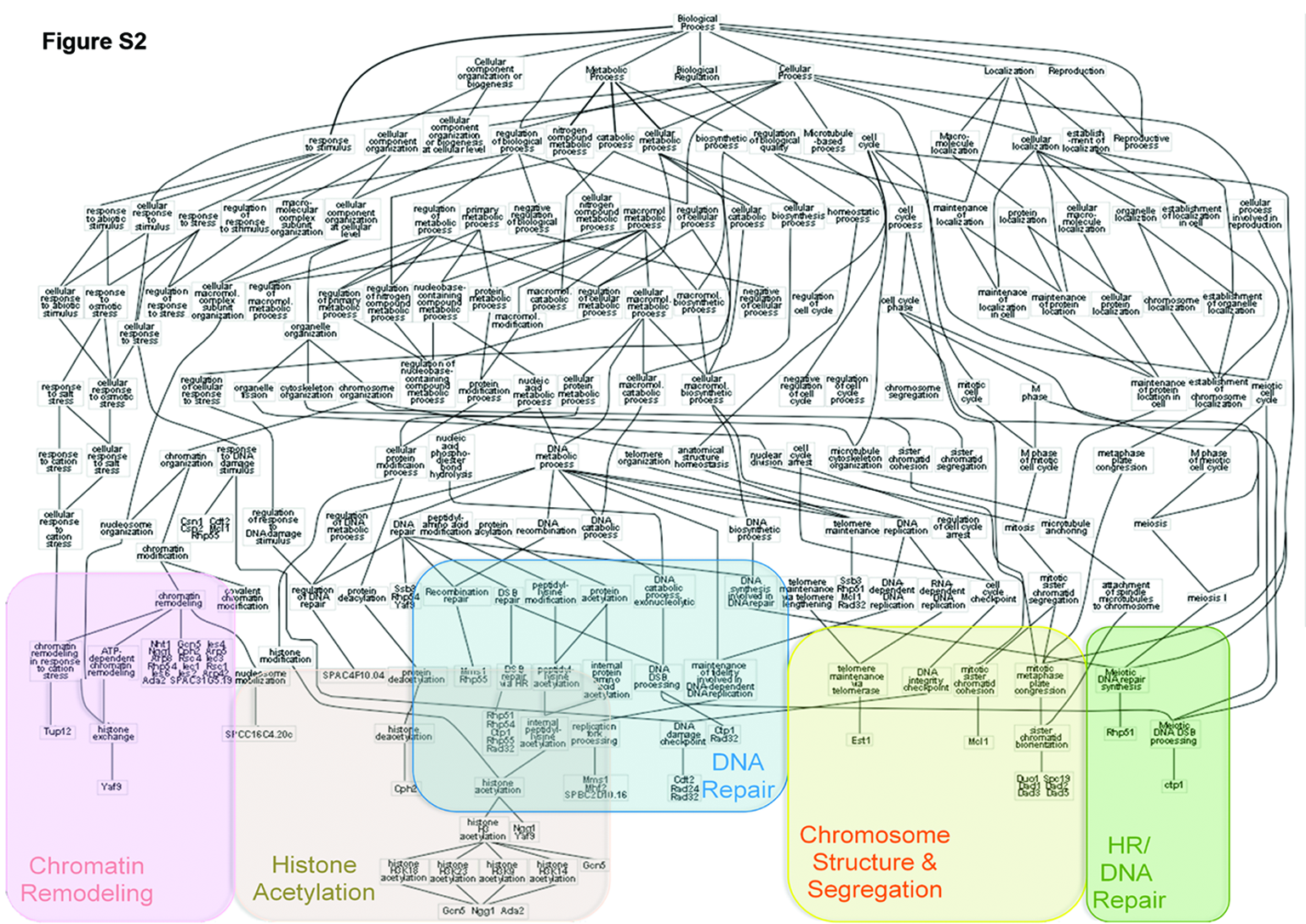

Supplement: Figure S2 — Ontological classification of DXR genes generated using GOLEM. All highly significant DXR genes were allocated by GOLEM to pathways related to regulation of DNA damage response, homologous recombination, chromosome structure maintenance and chromosome segregation, chromatin remodeling and histone acetylation. The genes depicted in this chart were enriched in several distinct macromolecular complexes, namely the microtubule connector at kinetochore DASH (Duo1, Spc19, Dad1, Dad2, Dad3, Dad5), chromatin remodeler Ino80 (Nht1, SPCC16C4.02, Iec1, Ies2, Iec3, Ies4, Ies6, Arp5, Arp8), chromatin remodeler RSC (Rsc1, Rsc4, Arp42), SAGA (Gcn5, Ngg1, Ada2) and several HR factors (Rhp51, Rhp54, Rhp55). (TIF) [file pone.0055041.s002.tif]

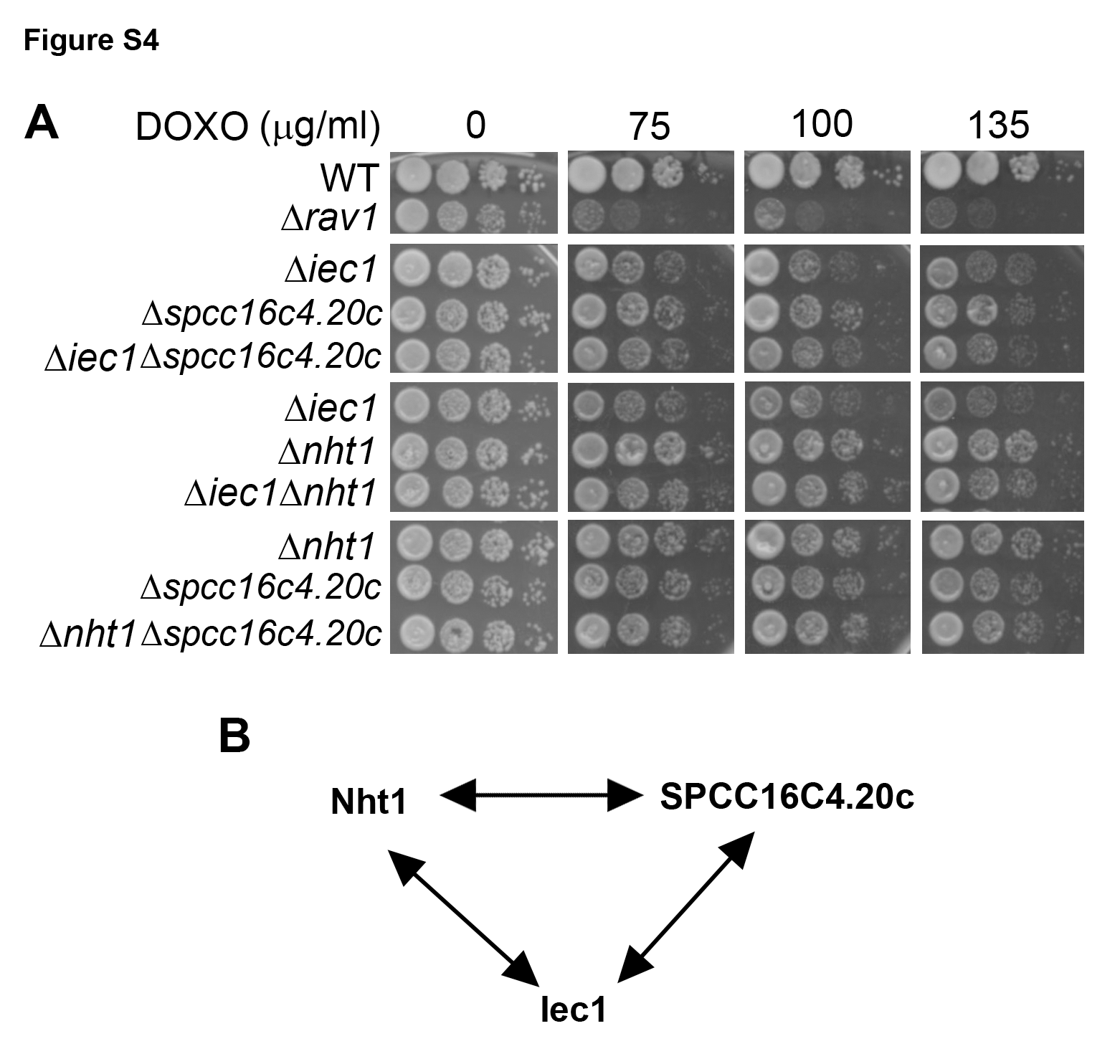

Supplement: Figure S4 — Genetic interaction between components of the Ino80 complex. Mutants of the Ino80 complex subunits showed no cumulative sensitivity to DOXO in double mutant combination over single mutants suggesting that they function in the same complex to regulate DOXO resistance. (A) Single and double mutants between Iec1, Spcc16c4. 20c and Nht1 were ten-fold serially diluted and spotted on plates incorporated with the indicated concentrations of DOXO. (B) Schematic representation of the close relationship between Iec1, Spcc16c4. 20c and Nht1. Double arrowhead lines depict no synthetic effect. (TIF) [file pone.0055041.s004.tif]

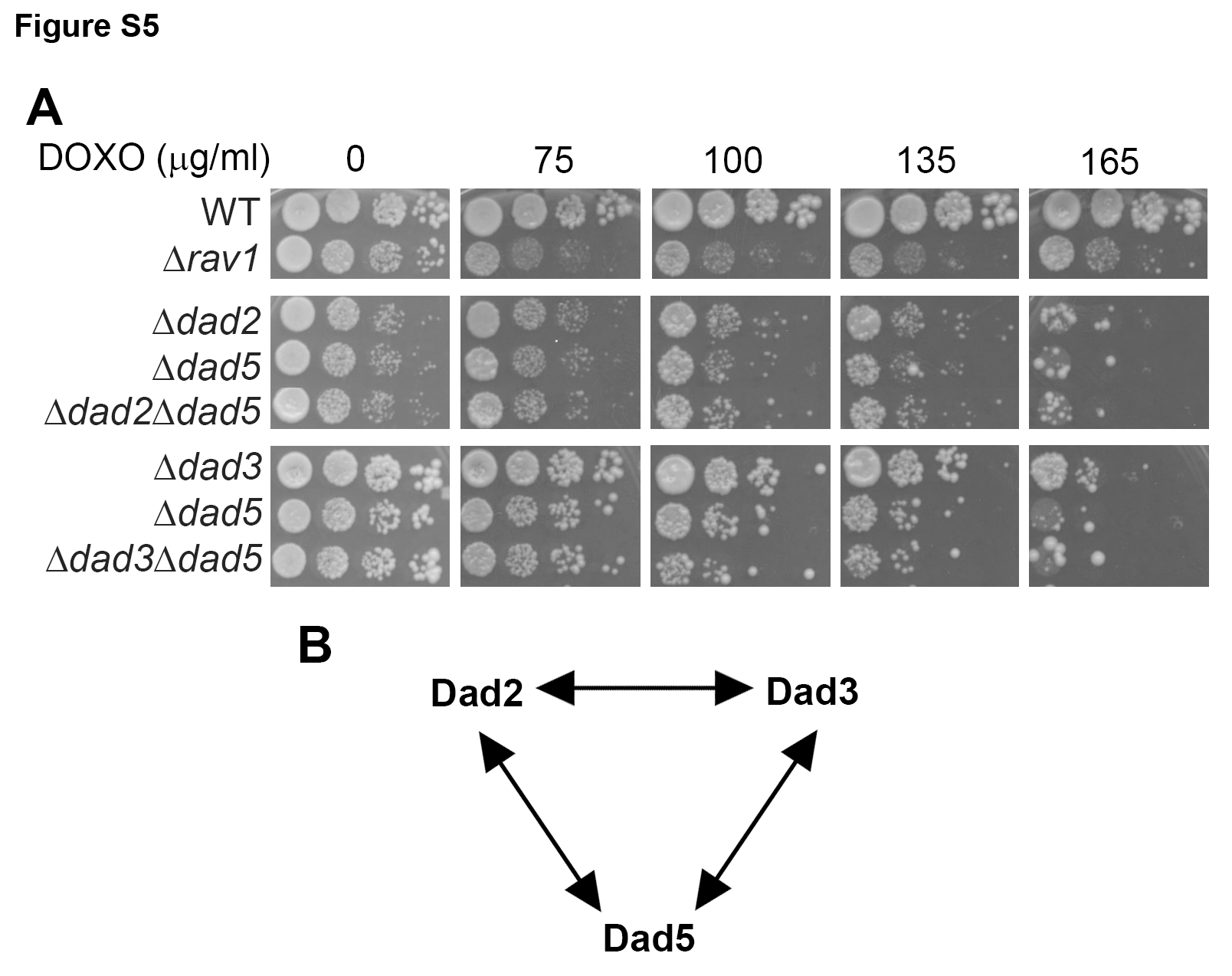

Supplement: Figure S5 — Genetic interaction between subunits of the DASH complex. Lack of cumulative DOXO hypersensitivity between mutants of different subunits of the DASH complex. (A) Single and double mutants between Dad2, Dad3 and Dad5 were ten-fold serially diluted and spotted on plates incorporated with the indicated concentrations of DOXO. (B) Schematic representation of the close relationship between Dad2, Dad3 and Dad5. Double arrowhead lines depict no synthetic effect. (TIF) [file pone.0055041.s005.tif]

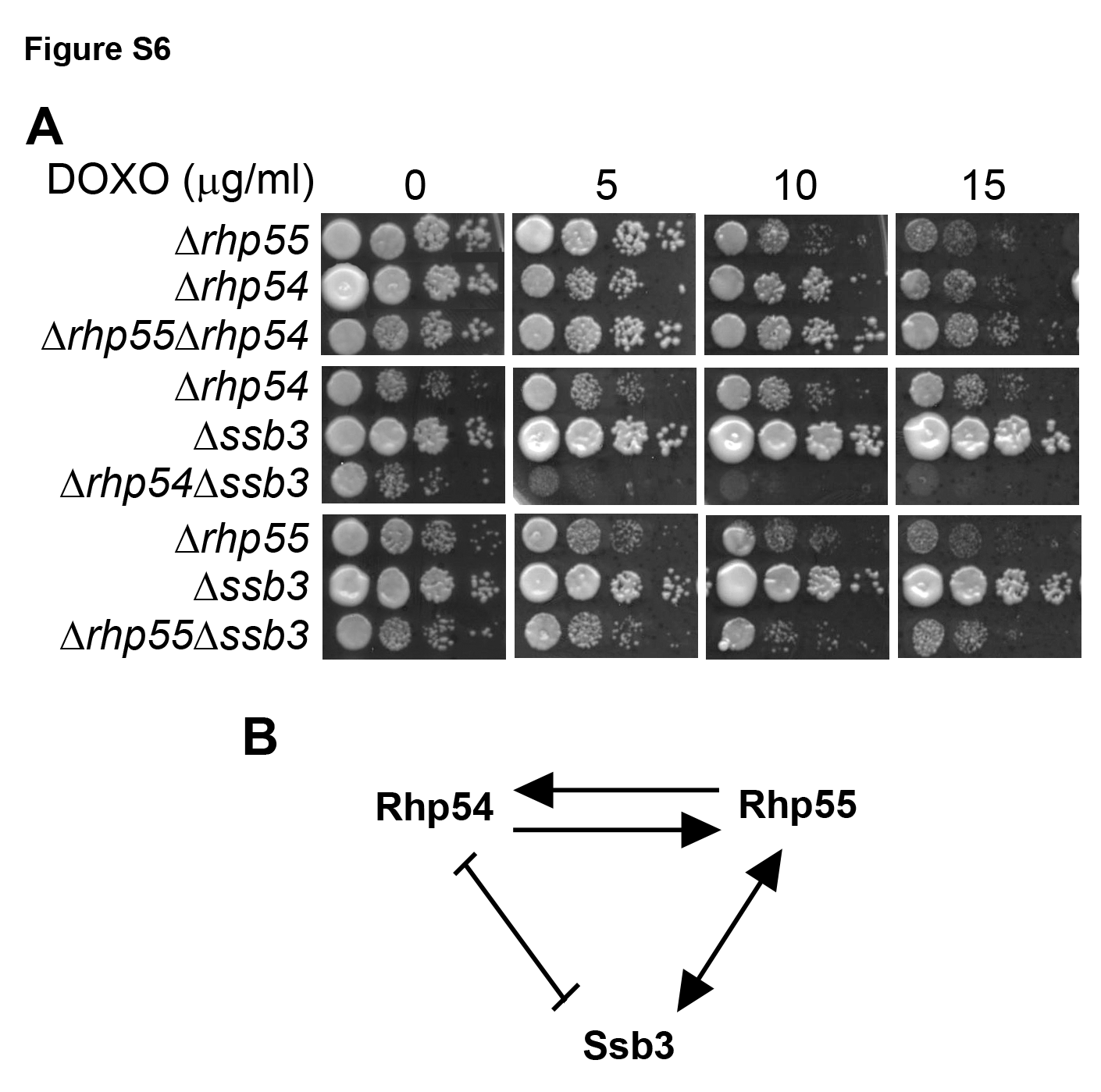

Supplement: Figure S6 — Genetic interaction between homologous recombination DXR genes. Genetic interaction between the single stranded DNA binding protein Ssb3 with the HR proteins Rhp54 and Rhp55 in DOXO. (A) Rhp54 and Rhp55 showed positive genetic interaction. Ssb3 was epistatic with Rhp55 suggesting that Ssb3 function in the same pathway with Rhp55. On the other hand, Δssb3Δrhp54 showed cumulative hypersensitivity over the single mutants. (B) Schematic representation of the close relationship between Ssb3, Rhp54 and Rhp55. Double arrowhead lines depict no synthetic effect. Single arrowhead represents synthetic suppression with the mutant pointed by the arrow being suppressed. Double blunt ended line represents synthetic growth defect. (TIF) [file pone.0055041.s006.tif]

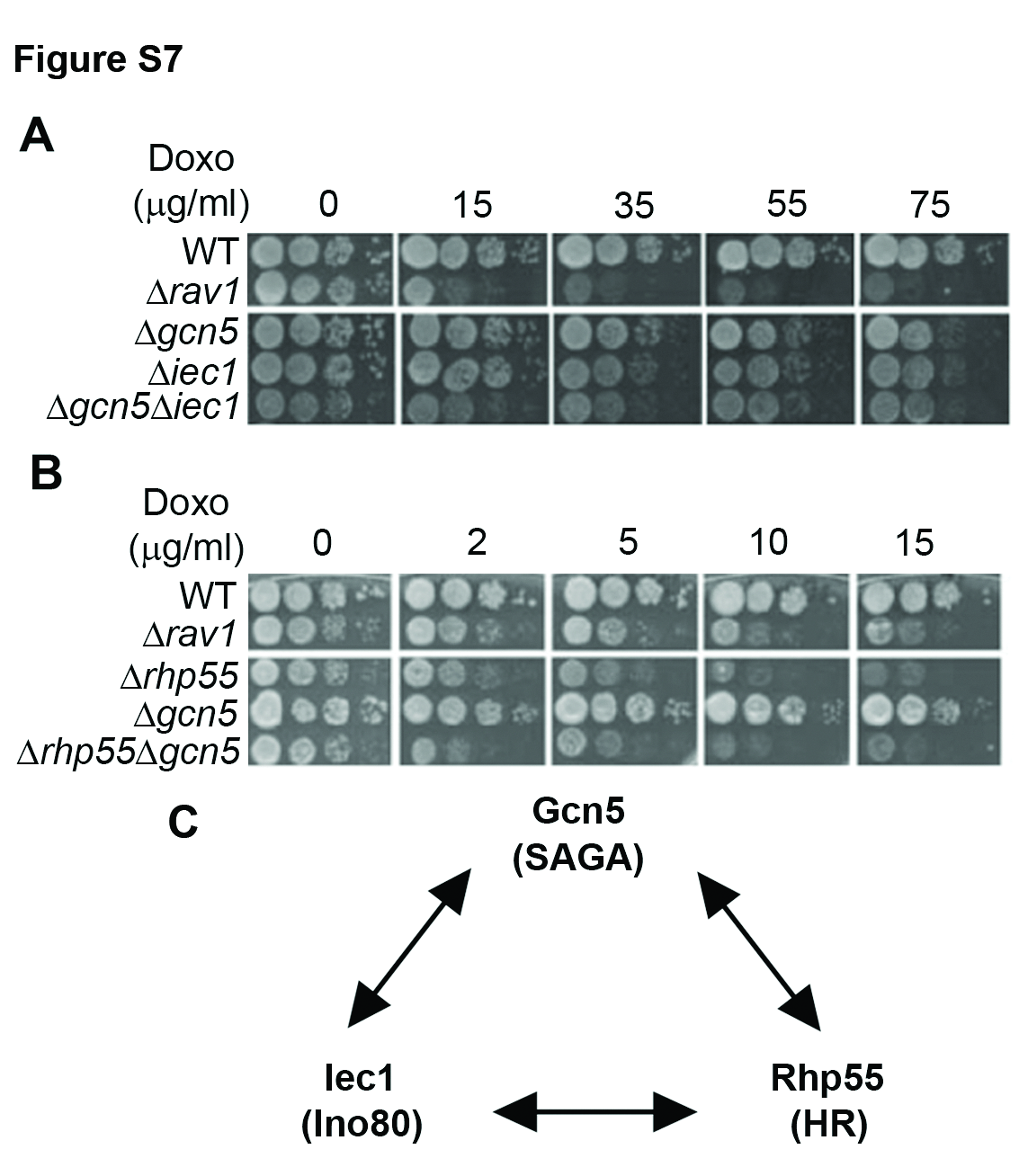

Supplement: Figure S7 — Homologous recombination factors, SAGA and Ino80 complex subunits formed a single epistatic group. (A) Mutants of the SAGA complex (Δgcn5), Ino80 complex (Δiec1) and homologous recombination factor (Δrhp55) were serially diluted and then manually spotted onto agar media containing the indicated concentrations of DOXO. Δgcn5Δiec1 double mutant showed no cumulative hypersensitivity relative to single mutants, indicating that these components of the three complexes were in the similar epistasis group. (B) DOXO hypersensitivity of Δrhp55Δgcn5 was equivalent to Δrhp55, which is the weaker of the two single mutants at the level of DOXO tested, showing that Rhp55 function in the similar epistatic group with Gcn5. (C) Schematic representation showing lack of synthetic growth defect (double arrowhead lines) between the mutants of SAGA (Δgcn5), Ino80 (Δiec1) and HR(Δrhp55) subunits. (TIF) [file pone.0055041.s007.tif]

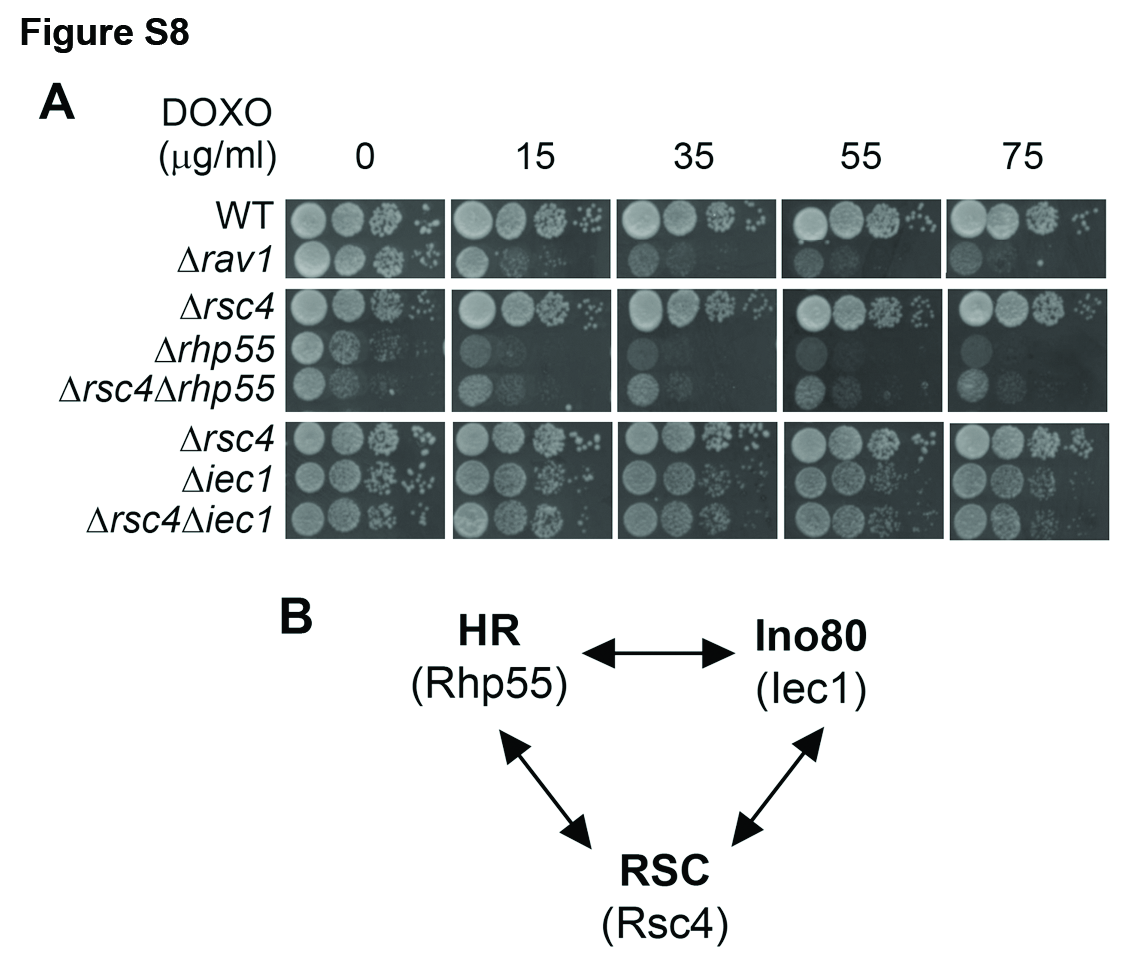

Supplement: Figure S8 — Homologous recombination, chromatin remodeler RSC complex and Ino80 complex subunits formed a single epistatic group. (A) Mutants of the SAGA complex (Δrsc4), Ino80 complex (Δiec1) and homologous recombination factor (Δrhp55) were tested as in Fig. S8. Δrsc4Δrhp55 and Δrsc4Δiec1 double mutant showed no cumulative hypersensitivity relative to single mutants. (B) Schematic representation of the relationship between Rsc4, Iec1 and Rhp55. Double arrowhead lines represent epistatic interaction accompanied by no cumulative increase in DOXO hypersensitivity relative to the single mutants. (TIF) [file pone.0055041.s008.tif]
