# Supplementary material for: Cellular Robustness Conferred by Genetic Crosstalk Underlies Resistance against Chemotherapeutic Drug Doxorubicin in Fission Yeast
Source: PLoS One. 2013 Jan 24;8(1):e55041. doi: 10.1371/journal.pone.0055041 (PMC3554685; doi:10.1371/journal.pone.0055041)
Supplement: Table S1 — DXR factors from fission yeast, budding yeast and human. (DOC) [file pone.0055041.s009.doc]

Table S1 DXR factors from fission yeast, budding yeast and human

| Biological processes | Hyper-sensitivity | *S. pombe* | *S. cerevisiae* | *H.sapiens* | *S. pombe* gene description |
| --- | --- | --- | --- | --- | --- |
| DNA repair | + | ssb3 | RFA3 | RPA3 | DNA replication factor A subunit Ssb3 |
|  | ++ | ctp1 | SAE2 | RBBP8 | CtIP-related endonuclease |
|  | ++ | mhf1 | YOL086W-A | MHF1 | FANCM-MHF complex subunit Mhf1 |
|  | ++ | mhf2 | YDL160C-A | STRA13 | FANCM-MHF complex subunit Mhf2 |
|  | ++ | mms1 | MMS1 | - | E3 ubiquitin ligase complex subunit Mms1 (predicted) |
|  | ++ | rad32 | MRE11 | MRE11A | Rad32 nuclease |
|  | ++ | rhp51 | RAD51 | RAD51 | RecA family recombinase Rhp51 |
|  | ++ | rhp55 | RAD55 | - | RecA family ATPase Rhp55 |
|  | ++ | rhp54 | RAD54 | RAD54L | Rad54 homolog Rhp54 |
| DNA damage checkpoint | + | rad24 | BMH1, BMH2 | 14-3-3 ε | 14-3-3 protein Rad24 |
| Chromatin remodeling | + | est1 | EST1 & EBS1 | SMG6 | Telomerase regulator Est1 |
|  | + | ies6 | IES6 | - | Ino80 complex subunit Ies6 |
|  | + | arp5 | ARP5 | - | actin-like protein Arp5 |
|  | + | iec3 | - | - | Ino80 complex subunit Iec3 |
|  | + | ies4 | IES4 | - | Ino80 complex subunit Ies4 |
|  | + | rsc1 | RSC1 & RSC2 | PBRM1 | Chromatin remodeling complex RSC, subunit Rsc1 |
|  | + | yaf9 | YAF9 | YEATS4 | YEATS family histone acetyltransferase subunit Yaf9 |
|  | ++ | arp8 | ARP8 | - | actin-like protein Arp8 |
|  | ++ | arp42 | ARP4 | ACTL6B | SWI/SNF and RSC complex subunit Arp42 |
|  | ++ | ies2 | IES2 | - | Ino80 complex subunit Ies2 |
|  | +++ | cph2 | *RCO1* | - | Clr6 histone deacetylase associated PHD protein-2 Cph2 |
|  | +++ | iec1 | - | - | Ino80 complex subunit Iec1 |
|  | +++ | SPAC31G5.19 | YTA7 | ATAD2 | ATPase with bromodomain protein (predicted) |
|  | +++ | SPCC16C4.20c | NHP10 | INO80E | Ino80 complex subunit (predicted) |
|  | ++ | nht1 | - | - | Ino80 complex HMG box protein Nht1 |
|  | ++ | rsc4 | RSC4 | PBRM1 | RSC complex subunit Rsc4 |
| Transcription-related | + | gcn5 | GCN5 | KAT2A | SAGA complex histone acetyltransferase catalytic subunit Gcn5 |
|  | + | rpa12 | RPA12 | ZNRD1 | DNA-directed RNA polymerase complex I subunit Rpa12 |
|  | ++ | yox1 | YOX1; YHP1 | - | MBF complex negative regulatory component Yox1 |
|  | + | ada2 | ADA2 | TADA2A | SAGA complex subunit Ada2 |
|  | + | php3 | HAP3 |  | CCAAT-binding factor complex subunit Php3 |
|  | ++ | ngg1 | NRM1 | - | SAGA complex subunit Ngg1 |
|  | +++ | nrm1 | *NGG1* | TADA3 | negative regulator of MBF |
|  | +++ | tup12 | *TUP1* | - | transcriptional corepressor Tup12 |
| Translation-related | + | SPBC19G7.10c | PAT1 | PATL1 | topoisomerase II-associated deadenylation-dependent mRNA-decapping factor (predicted) |
|  | + | SPAC6G9.14 | PUF4 & MPT5 | - | RNA-binding protein (predicted) |
|  | ++ | dph2 | DPH2 | DPH1 | diphthamide biosynthesis protein (predicted) |
|  | +++ | sce3 | *TIF3* | EIF4B | translation initiation factor (predicted) |
| Chromosome segregation | + | spc19 | SPC19 | - | DASH complex subunit Spc19 |
|  | + | dad1 | *DAD1* |  | DASH complex subunit Dad1 |
|  | + | duo1 | DUO1 | - | DASH complex subunit Duo1 |
|  | ++ | dad5 | HSK3 | - | DASH complex subunit Dad5 |
|  | + | ase1 | ASE1 | PRC1 | antiparallel microtubule cross-linking factor Ase1 |
|  | ++ | dad2 | DAD2 | - | DASH complex subunit Dad2 |
|  | +++ | dad3 | DAD3 | - | DASH complex subunit Dad3 |
|  | ++ | mcl1 | CTF4 | WDHD1 | DNA polymerase alpha accessory factor |
| dNTP metabolism | + | ccr4 | CCR4 | CNOT6L | CCR4-Not complex subunit Ccr4 (predicted) |
|  | + | caf1 | POP2 | CNOT7 | CCR4-Not complex CAF1 family ribonuclease subunit Caf1 |
|  | + | cdt2 | UTP15 | - | WD repeat protein Cdt2 |
|  | + | csn2 | - | COPS2 | COP9/signalosome complex subunit Csn2 |
|  | ++ | csn1 | - | - | COP9/signalosome complex subunit Csn1 |
|  | + | ada1 | AMD1 | AMPD2 | adenosine deaminase Ada1 (predicted) |
|  | + | SPAC2F3.11 | PPX1 | PRUNE | exopolyphosphatase (predicted) |
|  | ++ | SPBC651.07 | - | - | COP9/signalosome associated, sequence orphan |
| Membrane transporter | + | npp106 | NIC96 | NUP93 | nucleoporin Npp106 |
|  | ++ | apl6 | APL6 | - | AP-3 adaptor complex subunit Apl6 (predicted) |
|  | +++ | rav1 | RAV1 | - | RAVE complex subunit Rav1 |
|  | +++ | SPCC18.02 | nil | SLC18A2 | membrane transporter (predicted) |
|  | +++ | vph2 | VPH2 |  | endoplasmic reticulum membrane involved in assembly of the V-ATPase |
|  | + | erd2 | ERD2 | TXNDC17 | HDEL receptor (predicted) |
|  | + | vps35 | VPS35 | VPS35 | retromer complex subunit Vps35 |
|  | + | vps901 | VPS9 | RABGEF1 | guanyl-nucleotide exchange factor Vps901 (predicted) |
|  | ++ | apl5 | APL5 | AP3D1 | AP-3 adaptor complex subunit Apl5 (predicted) |
|  | ++ | SPAC2C4.05 | ERV15 & ERV14 | CNIH4 | ER vesicle integral membrane protein involved in endoplasmic reticulum membrane protein involved in assembly of the V-ATPase (predicted) |
|  | +++ | pmd1 | STE6 | ABCB1 | leptomycin efflux transporter Pmd1 |
| Signal transduction | + | git1 | - | - | C2 domain protein Git1 |
|  | + | SPAC4F10.04 | RRD1 | PPP2R4 | protein phosphatase type 2A, intrinsic regulator (predicted) |
|  | ++ | git5 | - | - | heterotrimeric G protein beta subunit Git5 |
| Mitochondria related | + | dps1 | COQ1 | PDSS1 | decaprenyl diphosphate synthase subunit Dps1 |
|  | + | SPAC823.10c | YDL119C | SLC25A38 | mitochondrial carrier with solute carrier repeats (predicted) |
|  | + | SPAC9E9.09c | - | - | aldehyde dehydrogenase (predicted) |
|  | + | SPCC1672.04c | COX19 | COX19 | mitochondrial copper chaperone (predicted) |
|  | + | SPCC1840.09 | YLR290C | - | NAD dependent epimerase/dehydratase family protein |
|  | ++ | coq2 | YDL119C | COQ2 | para-hydroxybenzoate--polyprenyltransferase |
|  | ++ | coq3 | COQ3 | COQ3 | hexaprenyldihydroxybenzoate methyltransferase Coq3 |
|  | ++ | coq4 | COQ4 | COQ4 | ubiquinone biosynthesis protein Coq4 (predicted) |
|  | ++ | coq6 | COQ6 | COQ6 | monooxygenase Coq6 (predicted) |
|  | ++ | coq7 | CAT5 | COQ7 | ubiquinone biosynthesis protein Coq7 |
|  | + | coq10 | COQ10 | COQ10A,B | mitochondrial ubiquinone binding protein Coq10 |
|  | + | cox6 | COX6 | COX5A | cytochrome c oxidase subunit VI (predicted) |
|  | + | tom7 | TOM7 | TOM7 | mitochondrial TOM complex subunit Tom7 (predicted) |
|  | + | tim11 | TIM11 | - | F0-ATPase subunit E (predicted) |
|  | + | SPAC17H9.08 | LEU5 | SLC25A16 | mitochondrial coenzyme A transporter (predicted) |
|  | + | cbp6 | CBP6 | - | mitochondrial respiratory chain complex assembly protein Cbp6 (predicted) |
|  | +++ | ppr1 | - | - | mitochondrial PPR repeat protein P­­pr1 |
| Lipid metabolism | ++ | lcf1 | *FAA1* | ACSL4 | long-chain-fatty-acid-CoA ligase Lcf1 |
| Unknown sequence | + | SPAC29B12.08 | - | - | sequence orphan |
|  | + | SPBC2F12.12c | - | c19orf29 | human c19orf29 ortholog |
|  | + | SPBC16H5.13 | - | - | WD repeat protein, human WDR7 ortholog |
|  | + | mfm2 | - | - | M-factor precursor Mfm2 |
|  | ++ | SPBC17A3.05c | HLJ1 | - | DNAJ/DUF1977 DNAJB12 homolog |

Degree of hypersensitivity of the mutants to DOXO is depicted as ‘+’ signs: +++ as the most, ++ as medium and + as the least hypersensitive. Color of the ‘+’ indicates the concentration of DOXO at which the mutants were sensitive at. Red: 75µg/ml, green: 165µg/ml, and blue: 310µg/ml.
