# Supplementary material for: Cellular Robustness Conferred by Genetic Crosstalk Underlies Resistance against Chemotherapeutic Drug Doxorubicin in Fission Yeast
Source: PLoS One. 2013 Jan 24;8(1):e55041. doi: 10.1371/journal.pone.0055041 (PMC3554685; doi:10.1371/journal.pone.0055041)
Supplement: Figure S3 — Coenzyme Q biosynthesis genes were essential for DOXO resistance in fission yeast. Coq2, Coq4, Coq6, Coq7 and Dps1 were identified by GOLEM to function in the ubiquinone/coenzyme Q biosynthetic pathway. (PDF) [file pone.0055041.s003.pdf]

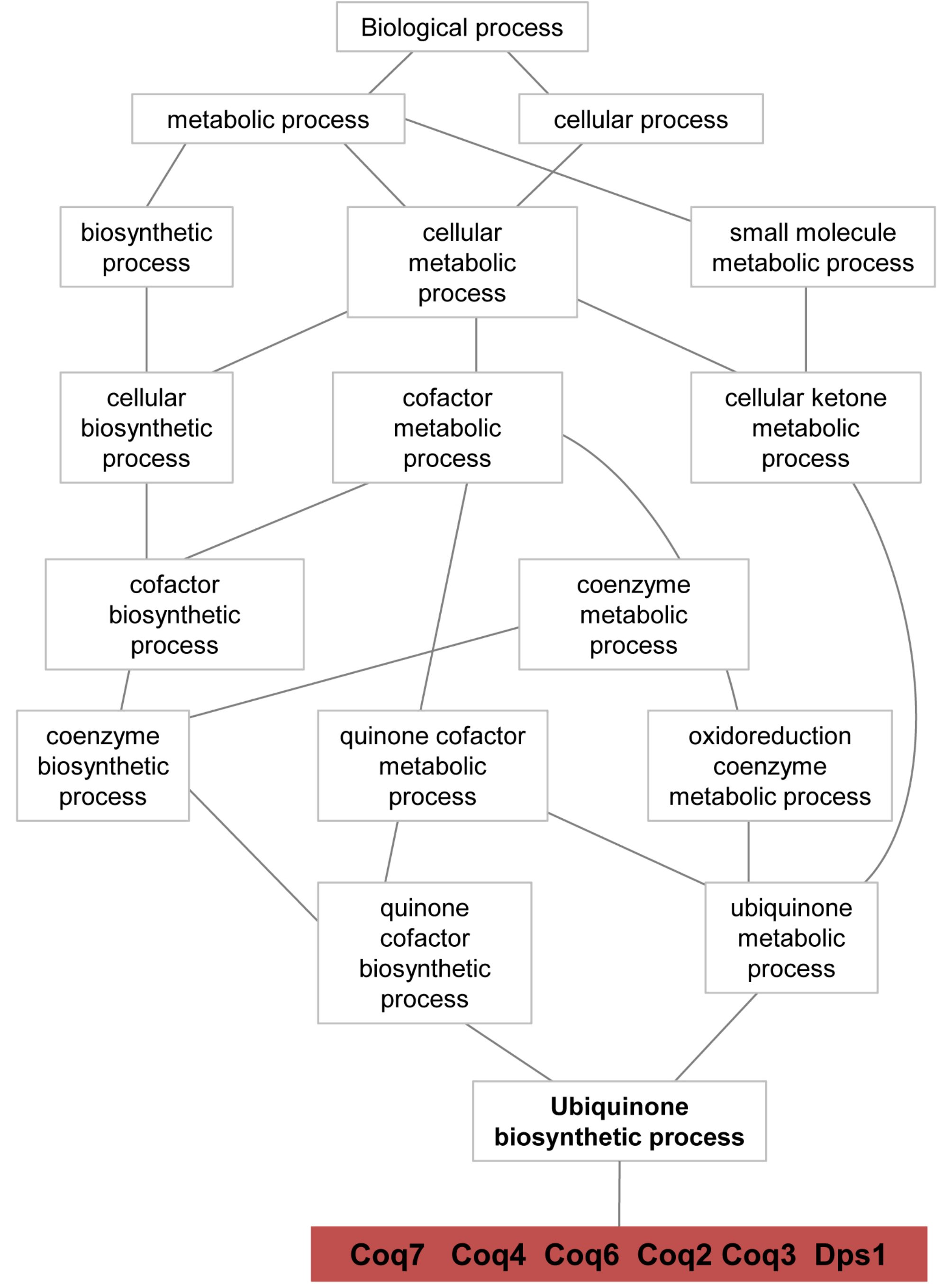

Biological process
metabolic process
cellular process
biosynthetic process
cellular metabolic process
small molecule metabolic process
cellular biosynthetic process
cofactor metabolic process
cellular ketone metabolic process
cofactor biosynthetic process
coenzyme metabolic process
coenzyme biosynthetic process
quinone cofactor metabolic process
oxidoreduction coenzyme metabolic process
quinone cofactor biosynthetic process
ubiquinone metabolic process
Ubiquinone biosynthetic process
Coq7 Coq4 Coq6 Coq2 Coq3 Dps1
